# The shadow model: how and why small choices in spatially explicit species distribution models affect predictions

**DOI:** 10.7717/peerj.12783

**Published:** 2022-02-14

**Authors:** Christian J. C. Commander, Lewis A. K. Barnett, Eric J. Ward, Sean C. Anderson, Timothy E. Essington

**Affiliations:** 1 Department of Biological Science, Florida State University, Tallahassee, Florida, United States of America; 2 School of Aquatic and Fishery Sciences, University of Washington, Seattle, Washington, United States; 3 Resource Assessment and Conservation Engineering Division, Alaska Fisheries Science Center, National Marine Fisheries Service, NOAA, Seattle, Washington, United States; 4 Conservation Biology Division, Northwest Fisheries Science Center, National Marine Fisheries Service, NOAA, Seattle, Washington, United States; 5 Pacific Biological Station, Fisheries and Oceans Canada, Nanaimo, British Columbia, Canada

**Keywords:** Species distribution model, Spatiotemporal model, Distribution shift, Range shift, Time series, Abundance estimation

## Abstract

The use of species distribution models (SDMs) has rapidly increased over the last decade, driven largely by increasing observational evidence of distributional shifts of terrestrial and aquatic populations. These models permit, for example, the quantification of range shifts, the estimation of species co-occurrence, and the association of habitat to species distribution and abundance. The increasing complexity of contemporary SDMs presents new challenges—as the choices among modeling options increase, it is essential to understand how these choices affect model outcomes. Using a combination of original analysis and literature review, we synthesize the effects of three common model choices in semi-parametric predictive process species distribution modeling: model structure, spatial extent of the data, and spatial scale of predictions. To illustrate the effects of these choices, we develop a case study centered around sablefish (*Anoplopoma fimbria*) distribution on the west coast of the USA. The three modeling choices represent decisions necessary in virtually all ecological applications of these methods, and are important because the consequences of these choices impact derived quantities of interest (*e.g*., estimates of population size and their management implications). Truncating the spatial extent of data near the observed range edge, or using a model that is misspecified in terms of covariates and spatial and spatiotemporal fields, led to bias in population biomass trends and mean distribution compared to estimates from models using the full dataset and appropriate model structure. In some cases, these suboptimal modeling decisions may be unavoidable, but understanding the tradeoffs of these choices and impacts on predictions is critical. We illustrate how seemingly small model choices, often made out of necessity or simplicity, can affect scientific advice informing management decisions—potentially leading to erroneous conclusions about changes in abundance or distribution and the precision of such estimates. For example, we show how incorrect decisions could cause overestimation of abundance, which could result in management advice resulting in overfishing. Based on these findings and literature gaps, we outline important frontiers in SDM development.

## Introduction

Human-induced global climate change and other anthropogenic activities are transforming ecosystems (Foley et al., 2005; Walther, 2010; Doney et al., 2012). Species are altering their phenology (Cotton, 2003; Edwards & Richardson, 2004; Poloczanska et al., 2016), and many are shifting their distributions (Perry et al., 2005; Parmesan, 2006). Indeed, range shifts have been observed broadly across taxonomic groups (Hickling et al., 2006; Chen et al., 2011; Poloczanska et al., 2016), and these distribution shifts present many challenges for monitoring and management (Burrows et al., 2011). For example, transboundary exchange across political boundaries (*e.g*., Oremus et al., 2020) necessitates increased monitoring and coordination, and phenology shifts may require adjusting seasonal timing and geographic location of surveys, particularly if they drive distribution shifts (Chuine, 2010). Management and conservation decisions necessitate effective tools to estimate species distribution shifts in space or time and predict future responses.

Species distribution models (hereafter SDMs, but also known by their special cases as ecological niche models, occupancy models, or climate envelope models) in conjunction with climate and ocean models are the primary tools for describing past and current species distributions, determining the drivers of these patterns, and forecasting distribution shifts. There are a wide range of SDM approaches, including maximum entropy (*e.g*., Elith et al., 2011; Merow, Smith & Silander, 2013), generalized linear and additive models (*e.g*., Guisan, Edwards & Hastie, 2002) with or without hierarchical mixed effects (*e.g*., Thorson et al., 2015; Anderson & Ward, 2019), boosted regression trees (Elith, Leathwick & Hastie, 2008), and random forests (*e.g*., Barnett et al., 2019; Stock et al., 2020). Species distribution models often combine spatially-referenced data on species occupancy or abundance with environmental data to make inferences about habitat suitability and predicted distribution (Elith & Leathwick, 2009). Although SDMs have broad applications (see Elith & Leathwick, 2009; Guillera-Arroita et al., 2015; Robinson et al., 2017 for comprehensive reviews), recent literature has focused on their use for assessing potential shifts in species distributions in response to climate change (Distler et al., 2015; Thorson, Pinsky & Ward, 2016; Morley et al., 2018). Species distribution models have been used to examine shifts in distribution under climate change scenarios for hundreds of commercially important fish and invertebrates (Cheung et al., 2009), terrestrial mammals (Levinsky et al., 2007), plants (Kelly & Goulden, 2008), birds (Matthews et al., 2011), invasive species (Jarnevich & Stohlgren, 2009), and entire food webs (Leidenberger et al., 2015).

The use of SDMs in scientific literature has greatly increased in the past few decades (Guisan et al., 2013), particularly in the marine realm (Robinson et al., 2017), but this increasing wealth of information and approaches also brings common challenges. Robinson et al. (2017) provide an excellent framework and road map for guiding the SDM process in general. However, general issues remain that need to be addressed—including deciding the type of response to be modeled (presence-only, presence-absence, or abundance), selecting covariates, and specifying scale (extent and resolution; both spatially and temporally; Araújo et al., 2005; Connor et al., 2019). Moreover, it is unclear how trade-offs among different model choices should be navigated to select the best model for any particular application (*e.g*., generating unbiased estimates of density, estimating area occupied, forecasting changes in range edges).

While a number of papers have explored key challenges relating to the SDM process (including Briscoe et al., 2019; Norberg et al., 2019; Brodie et al., 2020; Muscatello, Elith & Kujala, 2021), here, we focus our efforts on spatially explicit SDMs that have become widely used in the marine sciences and ecology. These methods can be broadly differentiated from other correlative modeling approaches described above in that in addition to predicting observed data, they estimate a spatial process (spatial processes may consist of parameters controlling the spatial autocorrelation, for instance). Projecting the spatial process across the domain of the observed data allows one to construct spatial fields; because these fields are often estimated as random effects, they can be described as Gaussian random fields (Abrahamsen, 1997). In a regression setting, the spatial field can be incorporated alongside a design matrix of covariates, $g\left( {{u_s}} \right) = \; {{x}_s}{ b} + {\omega _s}$. The estimated field ${\bf \omega}$ is assumed to be ${\bf \omega} \sim {\rm MVN}\left( {0,{ {\bf \sum}}} \right)$ where the covariance matrix ${\bf \sum}$ represents the spatial covariance among points. These models are often described as semi-parametric models because rather than estimating all elements of ${\bf \sum }$, flexible covariance functions are used to model the relationship (*e.g*., Gaussian, Matérn), thereby reducing the number of estimated parameters (Shelton et al., 2014). Alternative options for reducing the dimensionality of spatial processes exist, including Conditional Autoregressive (CAR) and Simultaneous Autoregressive (SAR) models for areal or lattice data (Ver Hoef, Hanks & Hooten, 2018), but interpreting spatial correlation with these methods can be difficult because correlation is based on neighboring cells rather than continuous distance (Wall, 2004). For high dimensional georeferenced datasets with hundreds of observations per time step, estimation of ${\bf \sum}$ may still be computationally challenging. An approximation exists for these cases—rather than estimate the exact values of the spatial field at the locations of observations, predictive process models allow the spatial field to be approximated by a lower dimensional spatial representation at a smaller set of locations (knots). The estimated field at this subset may then be projected to the finer scale locations (Latimer et al., 2009).

For very large datasets, the Gaussian predictive process model may still lead to undesirable results, because while using a smaller number of knots may be computationally efficient, reducing the dimensionality too much will over smooth predictions of the spatial field. An alternative framework for high dimensional spatiotemporal models includes the Integrated Nested Laplace Approximation (INLA, Rue, Martino & Chopin, 2009) combined with Stochastic Partial Differential Equations (SPDE, Lindgren, Rue & Lindström, 2011; Miller, Glennie & Seaton, 2020). INLA is available in R (R Core Team, 2020) and allows for relatively fast estimation of spatial and non-spatial parameters in a Bayesian framework (Bakka et al., 2018). The SPDE approach requires constructing a spatial mesh as an approximation to the GMRF (Gaussian Markov random field) spatial field (Fig. S1; Rue, Martino & Chopin, 2009; Lindgren, Rue & Lindström, 2011; Righetto et al., 2018), which has a sparse precision matrix. Predictions at the knots of the triangulated mesh are used to interpolate predictions to other locations (Lindgren & Rue, 2015). Inference about spatial processes and trends in SDMs may be influenced by both the number and location of these knot values (Righetto et al., 2018); too few knots will not accurately approximate the spatial field (Righetto et al., 2018; Anderson & Ward, 2019) and may result in estimates that are biased (Thorson et al., 2021).

Given the rise of spatially explicit predictive process models in marine sciences and ecology over the last decade, there is a great need to understand how specific model choices affect inference. In addition to inference about parameters used to describe the spatial field, inference about derived quantities can be important for natural resource management. Within the context of fisheries for example, generating abundance indices that are accurate and precise is critical because these are a key fishery-independent data source for population assessment models used to determine conservation status and set exploitation rates (Hilborn & Walters, 1992).

**Figure 1:**
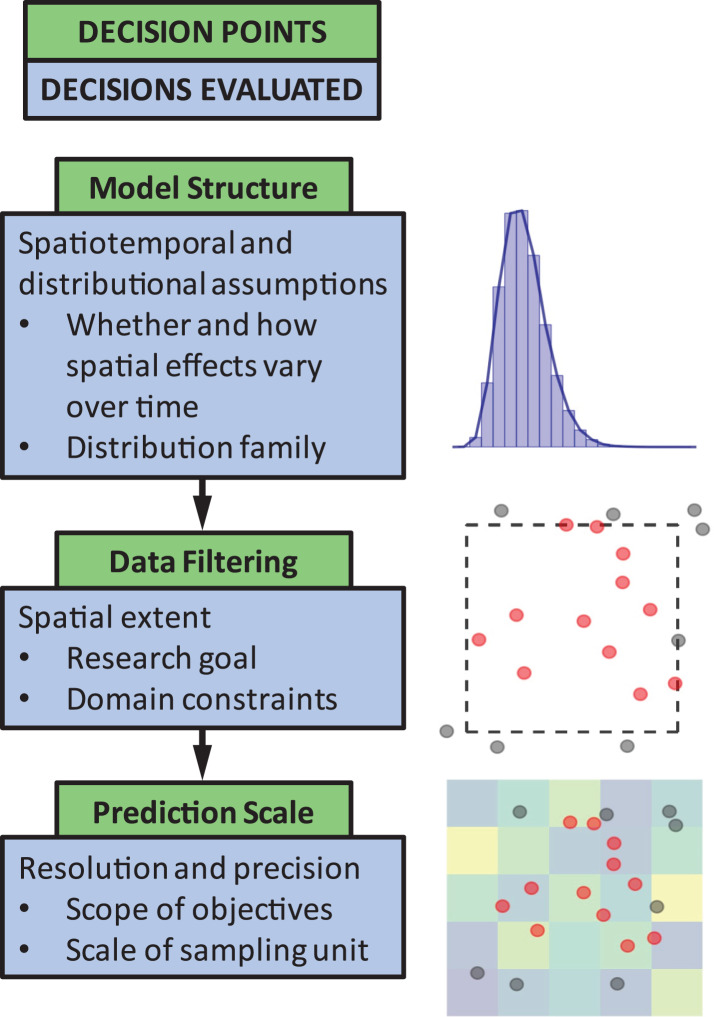
The three decision points in the species distribution modeling process evaluated in this paper.

The objective of this paper is to quantify the impact of three common decision points in these types of models (Fig. 1). The decision points we chose to highlight are seemingly small model choices that can greatly impact results, and thus, can lead to erroneous conclusions about population status or affect scientific advice to management decisions. These include (1) identifying the appropriate model structure, (2) choosing whether to filter data *a priori* based on spatial extent of occurrence (*e.g*., whether or not to include observed 0’s at the domain edge), and (3) selecting a spatial resolution for prediction. We outline and discuss these key decision points to guide SDM users by weaving together lessons from the literature and a real-world case study on the distribution of sablefish (*Anoplopoma fimbria*), a commercially valuable species found throughout the North Pacific Ocean. Though a marine application, this type of georeferenced survey data is common and has parallels in other terrestrial and aquatic systems. While the decision points chosen are specific to spatially explicit SDMs, these issues are also generally relevant to other SDM approaches (including generalized additive models). Our aim is to provide high-level advice regarding model structure and spatial scale considerations, and how to evaluate the consequences of these choices.

## Methods

### Data

Species distribution models require spatially referenced data, but the way in which space is represented varies among modeling approaches (data may consist of observations in spatial blocks, or observations may be associated with continuous locations in one or more dimensions). Here we focus on models that take geostatistical data as input. Geostatistical data may be collected during a single point in time, providing a snapshot of distributions, or they may be repeated at some regular interval (*e.g*., monthly or yearly surveys). In the latter case, the same sites may be revisited in each sampling interval, or sites may be spatially selected randomly. In addition to collecting observations of interest (*e.g*., species presence-absence or abundance), these types of surveys often collect data on potential covariates to include as predictors (*e.g*., habitat or environmental variables, or co-occurring species as proxies for effects of competition). Like simpler non-spatial models, choosing which covariates to include (and the scale of the covariates) requires forethought and can present challenges. For example, should one use raw data for covariates that exhibit a trend, or should anomalies or first-differenced values be used instead? How should one confront the problem of correlated or confounding covariates? At what scale should hypothesized covariates operate? For example, is population density determined by localized covariate values occurring in the same instance as the observed response, or by broader-scale processes due to non-local effects and lagged responses?

### Sablefish case study

As an example case study to illustrate the effect of model choices throughout this paper, we analyze a publicly available dataset of sablefish catch rates from scientific surveys in the Northeast Pacific Ocean. We chose sablefish as a representative of the commercially important groundfish community in the region because it is common and broadly distributed throughout the region (United States and Canadian waters) but also exhibits interannual variability, as well as a prominent environmental gradient in population density (with depth). The association with depth is largely ontogenetic; sablefish are long-lived predators that gradually move deeper with age and are most common along the continental slope (reviewed in Tolimieri et al., 2018; Haltuch et al., 2019). The sablefish dataset consists of a single survey conducted annually over a 16-year period on the continental shelf and upper slope (from 55–1,280 m depth) of the US West Coast from Cape Flattery, Washington to the USA-Mexico border as part of a fishery-independent scientific survey (the NOAA Fisheries US West Coast Groundfish Bottom Trawl Survey; Keller, Wallace & Methot, 2017). The survey was designed to estimate the abundance, size, and age composition of commercially and recreationally targeted groundfish species found in near-bottom habitats. Importantly, the fundamental methods of the survey: sampling intensity, sampling gear, seasonal and spatial coverage, and random stratified design have remained relatively constant within the period we analyze from 2003 to 2018 (data and maps of the survey area are publicly available at https://www.nwfsc.noaa.gov/data). Sablefish occur in a large proportion of observations (0.65) and are found throughout the spatial domain as their distribution extends southward to the southern tip of Baja California, Mexico, and northward to the Bering Sea and across the North Pacific to northern Japan (Eschmeyer & Herald, 1999).

### Statistical modeling

To develop a spatiotemporal model of sablefish, we constructed a model that can be seen as an extension of generalized linear mixed-effects models (GLMM), allowing for inclusion of both fixed and random effects. To ease in interpretation, we decompose total spatial variation into two components: a spatial field (a spatial process that is constant over time) and spatiotemporal fields (spatial variation that is unique to each time step). This approach has been widely used, especially in the context of estimating species’ densities, and has been shown to increase precision of estimated trends (Thorson et al., 2015), which is the focus of many applications in fisheries and ecology.

The generic spatiotemporal GLMM can be written as:

$g\left( {{u_{s,t}}} \right) = \;{x_{s,t}}b + {\omega _s} + {\varepsilon _{s,t}}$

where $g(\cdot)$ represents a link function (*e.g*., logit function for a binomial presence-absence model, log function for a model of abundance with a response distribution of counts or positive values, including Poisson, Negative Binomial, Gamma, Lognormal, *etc*.), and ${u_{s,t}}$ represents the expectation at location *s* and time *t*. The symbol ${{x}_{{s},{t}}}$ represents optional covariates modeled as fixed effects (*e.g*., an intercept, environmental or habitat variables, and seasonal or annual terms), and ***b*** represents a vector of estimated coefficients. As above, the symbol ${\bf \omega }$ represents a single estimated spatial field, with ${\omega _s}$ being the value at location *s*. We refer to ${\bf \omega }$ as the spatial component because it is constant over time (this can be interpreted as the mean spatial process shared across years). The term ${\varepsilon _{s,t}}$ represents spatiotemporal variability in the form of spatial random fields for each time step. The process describing ${\varepsilon _{s,t}}$ is flexible in that it can be omitted from the model (leaving a model with a spatial but no spatiotemporal component), may be independently estimated for each time step, or modeled with an autoregressive process (allowing hotspots to persist through time; Thorson et al., 2015; Ward et al., 2018; Anderson & Ward, 2019).

### Model choices specific to the sablefish case study

For the application of SDMs to sablefish throughout this paper, we decided *a priori* to include depth as a covariate predictor of density. Previous applications to groundfish density have shown community structure and within-species size distributions often vary with depth (Methot & Stewart, 2005; Hamel, 2007; Tolimieri, 2007), and depth—and the many environmental variables that covary with it (*e.g*., temperature)—is likely important in determining groundfish distribution and certain biological processes such as growth (Hamel, 2007; Johnson, Thorson & Punt, 2019). Compared to other covariates in studies of US West Coast groundfishes, depth was found to explain the greatest variation in density (Shelton et al., 2014). The form of depth included in our models was as a quadratic (Shelton et al., 2014; Thorson et al., 2015) since the quadratic shape rendered ecologically realistic environmental relationships and improved model fit over a linear fit. Other approaches to model this relationship could include splines or random walks with binned depth values (Webster et al., 2020).

### Estimation

Several recent advances have allowed for maximum likelihood estimation to be done using Template Model Builder (Kristensen et al., 2016) using SPDE matrices output by INLA. This approach often allows for faster estimation than using INLA for model fitting (Osgood-Zimmerman & Wakefield, 2021) and allows for additional model flexibility (*e.g*., Barnett, Ward & Anderson, 2021). R packages facilitating such estimation with pre-specified models include VAST (Thorson, 2019) and sdmTMB (Anderson, Keppel & Edwards, 2019; Anderson et al., 2020). For our sablefish case study, we used the latter approach for all model fitting (https://github.com/pbs-assess/sdmTMB). This package is useful for large and complex datasets that can be computationally taxing (Anderson et al., 2020), it allows for many different response distributions, and optionally includes spatially (*e.g*., Barnett, Ward & Anderson, 2021) and temporally (*e.g*., English et al., 2022) varying coefficients. While not thoroughly explored in this paper, practitioners could use model selection tools to compare alternative models (*e.g*., with or without spatiotemporal processes, time-varying depth effect, *etc*.). We encourage readers interested in using model selection tools to carefully consider how their questions and goals may dictate the best approach to model selection (Tredennick et al., 2021).

### Model validation

A common goal of predictive process SDMs, like any other statistical model, is to identify model features that improve predictive ability. Widely used model selection tools, like AIC, are computationally convenient—but not wholly appropriate for mixed effects models (Liang, Wu & Zou, 2008). With large datasets, cross-validation offers an opportunity to evaluate the out-of-sample predictive ability of a given model. There are many choices that can be made to select the folds or partitions for a given dataset. These folds may be selected randomly, in spatial blocks to preserve autocorrelation (Valavi et al., 2019), or other approaches specific to the application. For our sablefish case study, we constructed folds based on latitude because there is typically more variability in groundfish densities as a function of latitude than other dimensions (Thorson & Ward, 2013). Using the quantiles of the distribution of latitude across all years, we constructed 5 blocks of approximately equal size and used these for k-fold cross validation, holding out each of the 5 folds as a test data set, and using the remaining folds as a training set. We used sdmTMB to generate predictions for each of the held out datasets in turn and calculated the log-likelihood of each observation (using the same likelihood distribution from the model fit to the training data). These out-of-sample measures of predictive density were then summed across observations and folds to generate total measures of predictive ability for each model.

### Sensitivity analysis

Given the spatiotemporal GLMM described above, we focused on the following three model choices that contribute to inference on parameters and derived quantities: (1) structure of the model, including covariate selection and treatment of spatiotemporal components, (2) spatial extent of data used for fitting, and (3) spatial resolution of predictions. To demonstrate the sensitivity to model resolution, we highlight how such a choice influences trends and scale of total population biomass estimates, derived from model predictions. We focus on biomass estimates (calculated as the sum of the local population densities extrapolated to the resolution of the prediction surface) here because this is a commonly used output of SDMs for population management (Thorson et al., 2015). To provide a metric for comparing differences in the distribution of abundance that would be estimated from each option within a decision point, we estimated the center of gravity (COG). Center of gravity is an important spatial indicator (*i.e*., statistic that characterizes spatial distribution) that describes the average geographical center of the population (Woillez, Rivoirard & Petitgas, 2009).

#### Model structure

Similar to choosing a response distribution for non-spatial models (McCullagh & Nelder, 1989; Zeileis, Kleiber & Jackman, 2008), choosing an appropriate response distribution is needed to represent the data outcomes for any SDM (Fig. 1). This decision is based on the type of data collected (*e.g*., discrete count, continuous, presence-absence), but also depends on the presence of zeros and dispersion in the data. Data with zeros and positive continuous values can be represented with a single distribution, such as the Tweedie distribution (Dunn & Smyth, 2005, 2008), or the model may separately estimate effects on the probability of occurrence and density in a delta- or hurdle framework (Pennington, 1983). For our SDMs developed for the sablefish case study, we used a Tweedie distribution (Dunn & Smyth, 2005, 2008) to account for zeros in our continuous response variable (catch per unit effort, CPUE, measured in kg per km^2^ swept by the sampling gear) since it has been shown to perform well in previous applications to such data (Anderson, Keppel & Edwards, 2019; Barnett, Ward & Anderson, 2021; Thorson et al., 2021; English et al., 2022). While the majority of hurdle models construct separate spatial fields for presence-absence and positive biomass density (*i.e*., processes assumed to be independent), an advantage of using the Tweedie distribution is that it involves modeling a single spatial field (processes dependent), simplifying interpretation (as an example of sharing elements across fields, see Martínez-Minaya et al., 2018).

The choice of response type and model structure should be informed by the data properties (*e.g*., whether units are in discrete number of fish or continuous catch per unit effort, whether or not the dataset contains 0’s) and the study objective. Additionally, the data generating process (*e.g*., knowledge of life-history characteristics) can inform how one accounts for spatiotemporal processes. Models can treat the spatial effects on distribution to be static over all time slices (spatial only model), or they can explicitly quantify how the spatial effects on distribution have changed through time (spatiotemporal model). Models that omit spatiotemporal effects may be well-suited for slow growing, long-lived species, or sedentary species with low dispersal rates; whereas models with spatiotemporal variation could be used, for example, to analyze hotspot persistence through time (Paradinas et al., 2020). If one chooses a spatiotemporal model, one must additionally decide whether spatiotemporal fields are estimated independently or by assuming temporal autocorrelation. This *a priori* decision depends largely on the study species and the hypothesized mechanisms that explain the species’ spatiotemporal patterns. For example, for patchily distributed sedentary or long-lived animals, a first order autoregressive (AR(1)) or random walk model could be used to model the spatiotemporal process. For other species, modeling these spatiotemporal fields as independent variation may be supported. For all of the alternative formulations of the spatiotemporal process, parameter estimates and traditional model selection techniques can be used to evaluate the data support for alternative models of the spatiotemporal process. For example, estimated values of an autoregression coefficient close to 0 in an AR(1) spatiotemporal model would give more credence to considering an alternative model with temporally independent fields (while values close to 1 would be supportive of a random walk model).

Evaluating the fit of predictive process SDMs and validating assumptions about structure involves considerations that are shared among many statistical models, but also includes others that are specific to—or particularly important for—spatial or spatiotemporal modeling. Examples of diagnostics for SDMs include the analysis of temporal or spatial autocorrelation in residuals (Cressie & Wikle, 2015; Ward et al., 2018), randomized quantile residuals (Dunn & Smyth, 1996; Hartig, 2021), one-step ahead residuals (Thygesen et al., 2017; Breivik et al., 2021), residuals from MCMC draws from the posterior (*e.g*., Rufener et al., 2021), and examining evidence of non-stationarity. The choice of additional metrics of fit often follows from previous decisions about model structure. For example, if delta or hurdle models are used to model variation in population density, one could use tools to evaluate the explanatory power of the presence-absence model (area under the curve, AUC, and extensions; Elith et al., 2006; Jiménez-Valverde, 2012). Alternatively, if the response is jointly modeled with a distribution that includes zeros and positive values (*e.g*., Tweedie, Negative Binomial, Poisson), other metrics of model fit will need to be used, including measures of predictive accuracy such as the log-score (Draper & Krnjajic, 2010).

As a demonstration of evaluating model structure in the sablefish case study, we performed a model comparison among four predictive process SDMs with different spatiotemporal processes. These models included (1) a model with only a spatial component and a covariate of depth, (2) spatiotemporal components that were independent and identically distributed over time ${\varepsilon _{\bf{t}}} \sim {\rm{MVN}}\left( {{\bf{0}},\sum } \right)$, (3) spatiotemporal effects modeled as an AR(1) process (see equations 10 and 11 in English et al., 2022), and (4) only a spatial component, without a depth covariate. To compare the out-of-sample predictive ability of these candidate models, we used the 5-fold cross validation procedure described above and calculated the predictive log-density for each model with the Tweedie likelihood. We evaluate the distribution of residuals resulting from simulating data from the model with parameters fixed at their maximum likelihood estimate, where predictions are conditional on the fitted random effects (Hartig, 2021). Additional modeling details and code to replicate these analyses are included in our GitHub repository: https://github.com/fate-spatialindicators/shadow-model.

#### Spatial extent of data

How far to extend the geographic domain of the model or range of predictions relative to the extent of observations is highly dependent on the application (Fig. 1). There may be a number of applications where making predictions beyond the range of observations may be a goal—examples include using SDMs to predict the spread of invasive species, making predictions into areas that are protected or otherwise physically inaccessible, quantifying range shifts related to warming environments, or identifying effects of habitat restoration for species that are locally extirpated (*e.g*., making predictions based on habitat suitability). Predictions beyond the edges of the sampling domain are expected to be imprecise, however there are cases where such predictions may still be useful (*e.g*., Pearson et al., 2007; Breivik et al., 2021). As an alternative example, there may be scenarios where the prediction grid should be smaller than the sampling domain: geographic features may constrain some species (freshwater lakes for terrestrial species, or islands in marine applications), or protected areas where sampling is not permitted. As an example of these restrictions, we modified our prediction grid for sablefish to exclude two regions that are not sampled by surveys due to their conservation status as protected areas (the Cowcod Conservation Areas, Yoklavich, Love & Forney, 2007).

In addition to making choices about the prediction grid, some applications of SDM predictive process models may also filter observations or restrict data prior to fitting. Filtering data may be needed in situations where the spatial domain of survey sampling varies year to year, but inference is about population change through time (using a subset of observations with a shared domain may be more appropriate). In other cases, it may be prudent to exclude observations that are thought to be beyond the species’ range (*i.e*., removing false absences). Such data filtering could be accomplished by removing observations falling outside an extent (or range of environmental covariates) defined by the limits of positive observations, or more restrictively, an extent defined by a convex hull or a chosen quantile of the kernel density estimate derived from positive responses. As a sensitivity analysis, we evaluated how data filtering based on latitude quantiles impacts inference about change in derived quantities for sablefish (specifically, trends in total biomass). Beginning with the full dataset, we calculated the univariate quantiles for latitude based on the full dataset and re-ran the same predictive process SDM (using the best fit model and same mesh resolution, in terms of the minimum distance between knot locations), dropping out 5–20% of the data at the northern and southern range edge. While we demonstrate the general sensitivity to the choice of data filtering at the range edge, if the objective is to determine what level of data filtering results in the best predictive skill, that would need to be evaluated by comparing predictions and observations on the same test data among approaches (*i.e*., that lying within the core of the species range).

#### Spatial resolution of predictions

A number of factors may affect the spatial resolution of predictions (Fig. 1), including the resolution of external covariates (Guisan et al., 2007). While the relationship between prediction resolution and uncertainty in predictive process models has not been addressed in many applications, one might expect intuitively that the resolution of the prediction surface from an SDM (*e.g*., a grid overlaid on the spatial domain) may affect inference by inflating or deflating estimates of uncertainty. Too fine of a spatial resolution could result in an underrepresentation of perceived uncertainty unless one can visualize uncertainty in the prediction at each location, a feat that is highly computationally demanding in predictive process SDMs. Too coarse of a resolution may increase uncertainty in derived products like total biomass estimates as this involves integrating over an increasing extent of modeled or unmodeled habitat covariates. A general rule of thumb for assessing an initial resolution is that it should typically be no finer than the scale of the sampling unit of the survey design. A rationale for making predictions at coarser scales may include trying to link the response of an SDM to environmental covariates predicted from global climate models, which often have a minimum resolution of ~10 km (Porfirio et al., 2014), to determine support for climate drivers of past changes or project future distribution changes. While a finer spatial prediction surface resolution may lead to improved model accuracy in some cases in both terrestrial and aquatic systems (*e.g*., Connor et al., 2019; Lowen et al., 2016), there are many caveats and much context-dependency to this relationship (Guisan et al., 2007 and references therein).

Beyond accuracy, another critical part of the predictive surface resolution decision point is consideration of the research objective. For example, in a study by Connor et al. (2019), the authors examined the effects of varying spatial scale on SDMs of giant pandas and found that changing spatial scale indeed impacted model accuracy. However, the predictive accuracy did not necessarily translate to ecological relevance, as the spatial scale that resulted in the highest accuracy was larger than individual panda home ranges and thus a larger environmental scale than they would correspond to Connor et al. (2019). Furthermore, Connor et al. (2019) used a modeling approach that ignores spatial autocorrelation, so it remains to be seen whether their conclusions hold in the context of the predictive process SDMs evaluated here.

For our case study of sablefish, we constrained the prediction grid to the same spatial resolution as the survey sampling frame (*i.e*., approximately 2.8 by 3.7 km). To do this, we used NOAA bathymetry data (https://www.ngdc.noaa.gov/mgg/coastal/crm.html) matched to the resolution of the trawl survey sampling units using bilinear interpolation. Additionally, to demonstrate the consequences of making predictions at too coarse of a resolution, we also constructed grids with cell sizes at 2–4 times larger than the sampling resolution. We focus on comparing estimates of biomass and center of gravity derived from models predicted to the original and 4× coarser resolution to evaluate the extremes of these scenarios.

## Results

### Model structure

Our evaluation of whether spatiotemporal effects are supported for modeling sablefish, and what form they should take if included, demonstrated that the model with the depth covariate and without any spatiotemporal components (model 1) had the highest predictive ability (Table 1). Therefore, we used model 1 as the base case for subsequent case-study analyses. All candidate models demonstrated slight overdispersion in the highest and lowest values (Fig. S2). The Matérn range parameter, controlling the distance at which two points are approximately independent, ranged from 22.83 to 67.00 across the folds in the model without spatiotemporal fields. Of the models with spatiotemporal components included, there was greatest support for the AR(1) model. Across folds, point estimates of the autocorrelation parameter ( $\varphi $) varied from 0.22 to 0.41 and the Matérn range parameter varied from 20.66 to 41.58.

**Table 1:** Predictive density (log-likelihood) for the four candidate models developed for the sablefish case study. All models include year as a fixed effect and spatial random effects. Five-fold cross validation was used to estimate the out-of-sample predictive density for each fold.

Model	Spatiotemporal effects	Covariates	Log density
1	–	depth, year	−68,301.36
2	IID	depth, year	−76,008.62
3	AR(1)	depth, year	−76,061.14
4	–	year	−82,156.34

**Figure 2:**
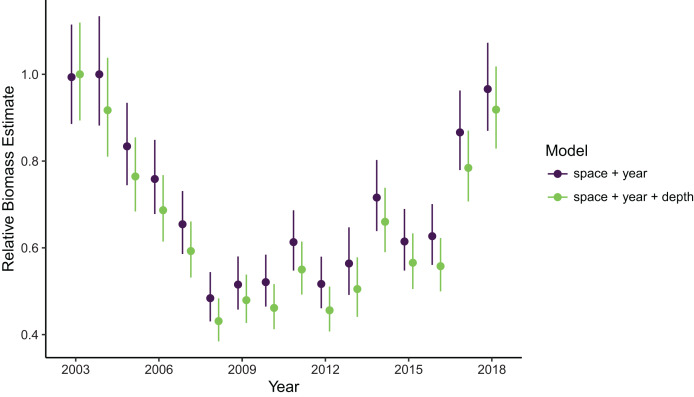
Relative sablefish biomass estimates (standardized to each time series’ maximum estimate) for each year (with 95% confidence intervals) compared between models with the best (model 1: space + year + depth) and worst (model 4: space + year) out-of-sample predictive skill.

**Figure 3:**
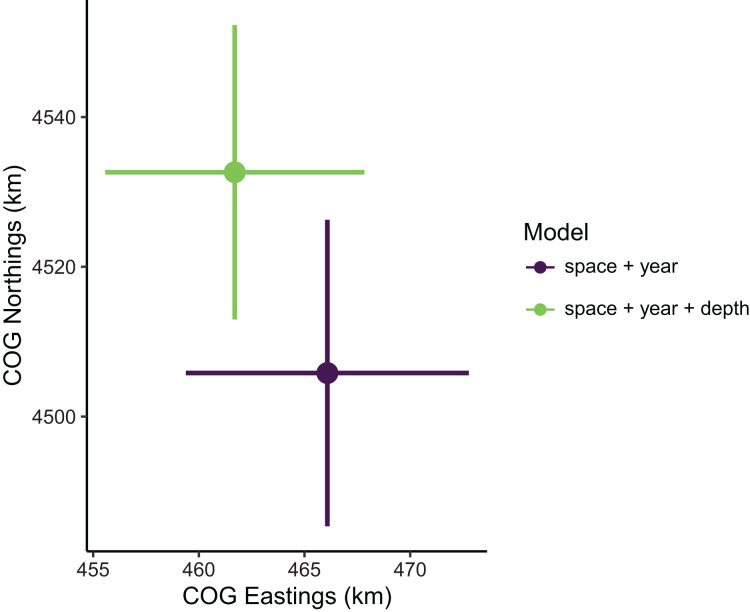
Sablefish center of gravity (COG) estimates compared between models with the best (model 1: space + year + depth) and worst (model 4: space + year) out-of-sample predictive skill. Points are COG and lines are 95% confidence intervals of Northings and Eastings (km).

Estimates of total population biomass and mean distribution (measured as the center of gravity or COG) differed between models with the best and worst out-of-sample predictive skill. Biomass estimates from the best model (model 1, including a spatial field and fixed effects of year and depth) were generally lower than estimates from the worst model (model 4, with the same structure as model 1 but without the depth covariate), with the exception of the first year of the time series where they were approximately equal (Fig. 2). Precision of biomass estimates was similar between models. Estimates of COG from the best model were further northwest than estimates from the worst model and precision was similar between models (Fig. 3). The above differences in outputs among models were low to moderate, and confidence limits on both biomass and COG estimates did overlap between models.

#### Spatial extent of data

**Figure 4:**
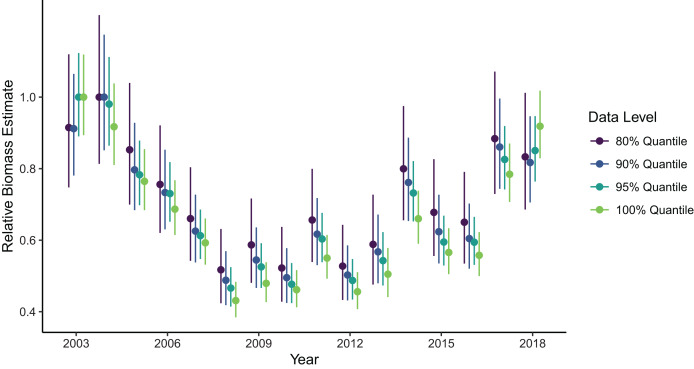
Relative sablefish biomass estimates (standardized to each time series’ maximum estimate) for each year (with 95% confidence intervals) for differing levels of data filtering constraining spatial extent (excluding observations outside a given quantile of the kernel density).

Biomass estimates were somewhat sensitive to the spatial extent of the data included in the model fit, with the greatest contrast in estimates being between model fits using data from the full domain as opposed to any level of truncation at the northern and southern domain limits (Fig. 4). Estimates made with the full dataset (*i.e*., no filtering at the domain edge) were generally lower than those made with the filtered data, except in the first and last year of the time series which were higher than filtered estimates. However, the range of confidence intervals on these estimates did overlap across all cases. Among estimates produced by the filtered datasets, removing more data from the domain edges generally produced higher estimates, with exception of the first and last years. The precision of biomass estimates decreased with increasing amount of data filtering, which is expected but possibly more pronounced than if it was being driven by changes in sample size alone.

**Figure 5:**
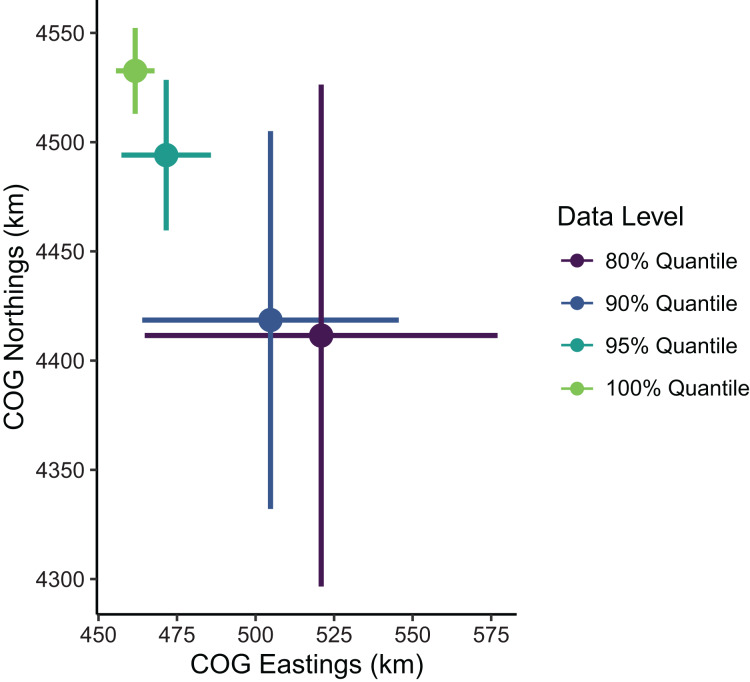
Sablefish center of gravity (COG) estimates for differing levels of data filtering constraining spatial extent (excluding observations outside a given quantile of the kernel density). Points are COG and lines are 95% confidence intervals of Northings and Eastings (km).

Estimates of the mean spatial distribution (indicated by the COG) were more sensitive to the spatial extent of data included than were biomass estimates. Greater extents of data filtering at the domain edge led to COG estimates that were much less precise and located further southeast compared to those with lesser extents of filtering (Fig. 5). In summary, the decision to limit the range extent should be well supported as this process can impact model estimates qualitatively. For the remaining examples in our SDM case study, we used the full data set without any filtering.

#### Spatial resolution of predictions

**Figure 6:**
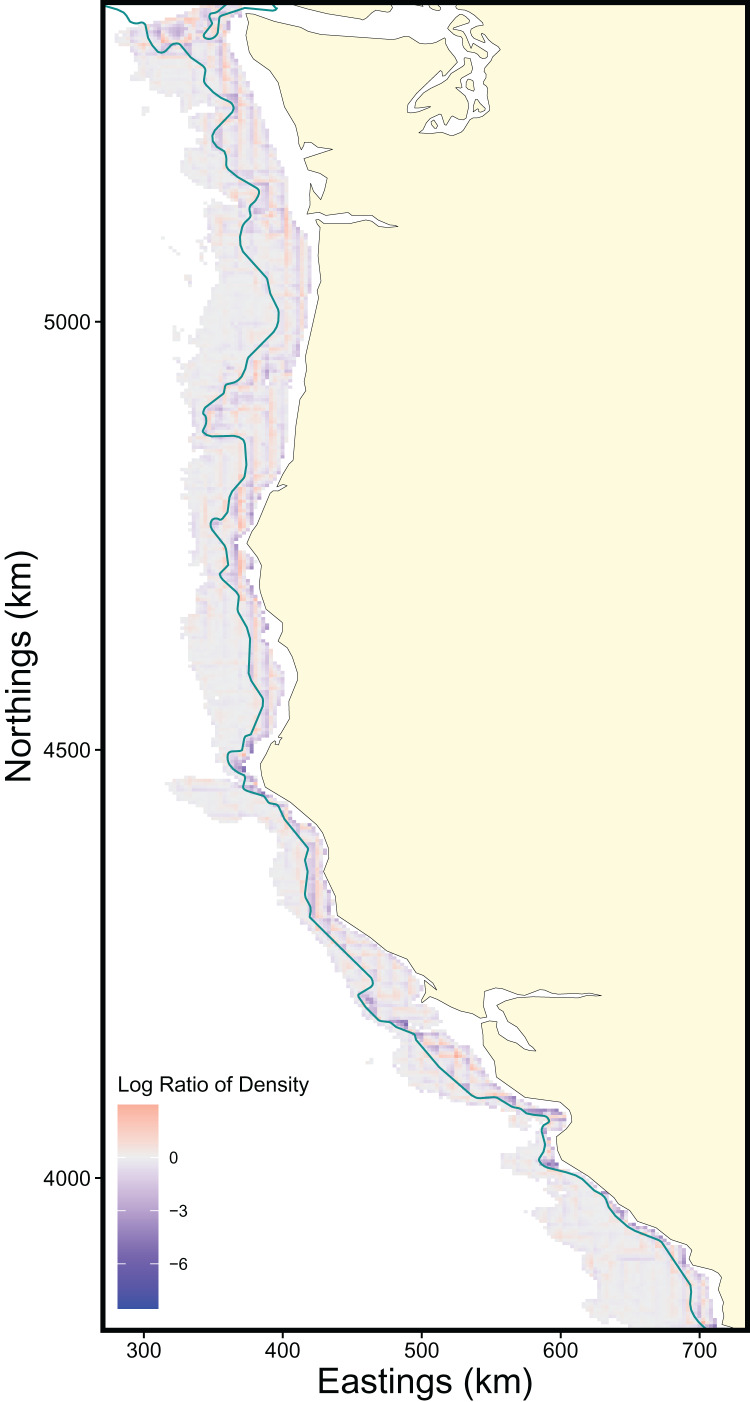
Map of differences in sablefish population density estimates (log-ratio) over the extent of the survey area between fine and coarse (4×) prediction surface resolutions for 2018, the most recent model year. Positive values (red) indicate higher predicted population density by the fine-resolution model and negative values (blue) indicate higher predicted density by the coarse-resolution model. Contour lines denote the 200 m isobath, which approximates the boundary between the continental shelf and slope.

**Figure 7:**
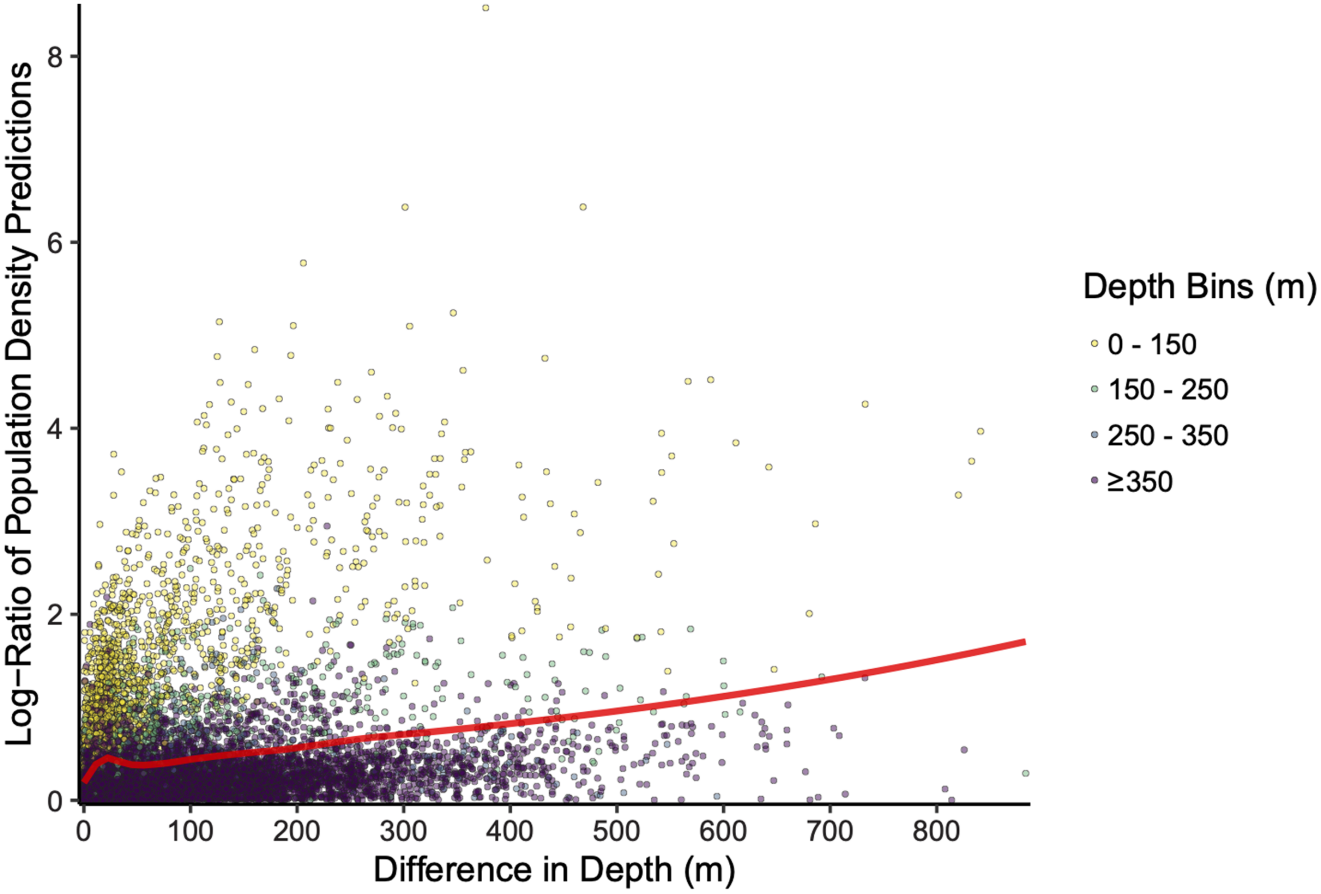
Differences in sablefish population density estimates (shown as the absolute value of the log-ratio to express the magnitude, but not the sign, of the differences) as influenced by differences in depth (m) between fine and coarse (4×) prediction surface resolutions for the most recent model year (*i.e*., 2018). Line denotes LOESS curve with a span of 0.5.

The scale of the prediction surface produced nuanced effects on predicted population densities in space. Comparing the SDM predictions in each grid cell from the fine scale prediction surface resolution (made at the same resolution as observed data) and coarse prediction surface (4× resolution) in space revealed the largest sensitivity to prediction scale occurred along the continental shelf and shelf break (~50–200 m depth), whereas deeper locations along the continental slope were less sensitive to prediction resolution (Fig. 6). This pattern of discrepancy between population density predictions by depth is because sablefish are most abundant in deep habitats, thus averaging our covariate of depth across a steep gradient such as the shelf break or canyon edge results in greater differences in depth observations at those locations, and these differences in depth drive the discrepancy in population density predictions (Fig. 7).

**Figure 8:**
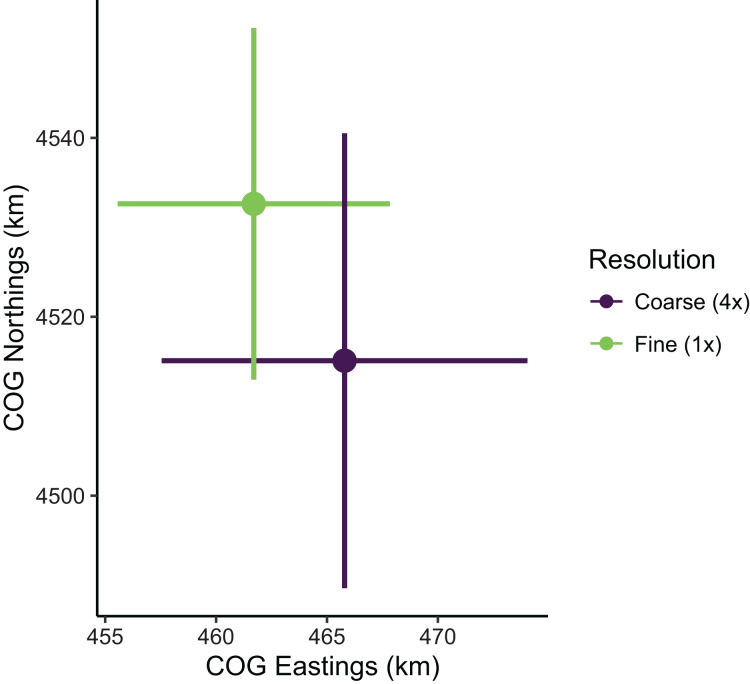
Sablefish center of gravity (COG) estimates for fine and coarse (4×) prediction surface resolutions. Points are COG and lines are 95% confidence intervals of Northings and Eastings (km).

Despite the apparent differences in local population densities, estimates of biomass and COG derived from these predictions had low sensitivity to the choice of prediction scale. Biomass estimates were not sensitive to the choice of prediction scale, as relative biomass estimates and their uncertainty were similar among prediction resolutions (Table S1; Fig. S3). However, COG estimates were somewhat sensitive to the choice of prediction scale (Table S2; Fig. 8). The center of gravity estimates from the coarse resolution (*i.e*., 4× the sampling resolution) were shifted slightly towards the southeast compared to estimates from the fine resolution, albeit with overlapping 95% confidence intervals (Fine: [4,513–4,552] Northings, [456–468] Eastings; Coarse: [4,490–4,541] Northings, [458–474] Eastings; all units in km; Table S2). Furthermore, the center of gravity was estimated with lower precision when using the coarse resolution (SE = 10.0 Northings, 3.1 Eastings) than fine resolution (SE = 13.0 Northings, 4.2 Eastings).

## Discussion

### Synthesis of recommendations for best practices for guiding modeling choices

In light of theoretical considerations, lessons learned from the literature, and illustrations through a worked example, we show how certain steps in the SDM process are key decision points influencing results and interpretation of SDMs. Though our focus is on spatial semi-parametric models, these choices are applicable to a variety of other SDM models:

1. Model family and structure: It is important to choose the appropriate model family and structure to be well-suited for the study species and the hypothesized underlying mechanisms that explain the species’ spatiotemporal patterns. Appropriate model structure need not involve throwing everything at the problem (*e.g*., using all potential covariates) and seeing what works, but rather using due diligence to select the appropriate predictors, and spatial and spatiotemporal structure.
2. Spatial extent of data and predictions: The spatial extent of data and predictions is highly dependent on the application of the SDM, but it is important to be mindful of the potential impact of data filtering and extrapolation of predictions on model outputs.
3. Spatial resolution of predictions: The spatial resolution of predictions should align with the research objective, but also typically be no finer than the scale of the sampling unit of the survey design.

Our case study application to sablefish illustrates that two of the three sensitivities (model structure and spatial domain of data used for the estimation) impact estimates of biomass trends and mean spatial distribution. While other studies have noted that the choice of prediction surface resolution is a critical decision that can affect precision and accuracy of predictions (Scales et al., 2017; Connor et al., 2019), we find that this did not carry over to predictions of total population size (here summarized by total annual biomass). Thus, we recommend researchers test sensitivity to prediction resolution within their own application, starting with a prediction surface resolution similar to that of the sampling unit that generated the input data. If non-local effects are expected (*e.g*., there are strong linkages to climate conditions at broader scales), models with coarser prediction resolution should be compared to this base case.

### Management implications of modeling choices

Seemingly minor decisions in the development of SDMs can influence the interpretation of model outputs used to inform conservation and management of fish and wildlife. From our sablefish example, filtering the observations in the model based on their spatial extent or use of an incorrect model structure could lead to the conclusion that the stock is more abundant than it truly is in many years, which could lead to catch recommendations that are too high. In addition, such oversights could lead to the inference that the population is distributed further southeast than it actually is. The potential influence of such deviations on expected management performance depends on the type of natural resource decision. For example, while fisheries management is becoming more spatial over time, spatial information or shifts are still rarely incorporated into stock assessment models (Goethel, Quinn & Cadrin, 2011; Berger et al., 2017). Conversely, in fish and wildlife conservation, management often considers distribution shifts in policy decisions—managers are sometimes interested in changes in distribution of habitat suitability for the purposes of managed relocation, or simply to prioritize locations for protected areas or habitat restoration (Guisan et al., 2013). Due to these differences, we will outline the potential major consequences of SDM decisions on these two fields by separately addressing the topics of abundance indices and distribution shifts.

Species distribution modeling decisions affecting abundance index scale (*i.e*., consistent bias over time) are less likely to influence management performance in this context than decisions affecting the trends, relative magnitude of temporal variability, or uncertainty in abundance estimates because abundance indices are typically treated as relative rather than absolute. However, in fisheries assessments where the index is the primary source of information, abundance indices are often treated as absolute, and in these cases the correct choice of spatial extent of the data and model structure becomes critical as biases resulting from model choices (this study; Thorson et al., 2021) can lead to poor catch recommendations and subsequent under- or over-fishing (the latter of which would be possible if biomass estimates were based on the worst performing model in the sablefish case study and the stock was close to limit reference points). In addition to the scale of the abundance index, we found that abundance was estimated with greater uncertainty as more data was filtered out at the extremes of the spatial domain. This increase in uncertainty can be problematic because it becomes more difficult to detect changes in abundance over time. For fisheries assessments that integrate multiple data sources, the coefficient of variation of abundance estimates is often used to weight data across years and data types (*e.g*., Francis, 2011). Thus, model choices similar to those in our sablefish case study may have the result of downweighting the abundance index relative to other data sources (each with their own biases, *e.g*., fishery-dependent data).

In the context of wildlife management and conservation, the reliability of predicted species distributions is perhaps just as critical to management performance as abundance estimates, thus modeling choices affecting indicators of change in core and limits of distributions can be highly influential. For example, erroneous estimates of the distribution of habitat suitability or population density could lead to failure by misrepresenting the benefits of habitat restoration or managed relocation by overestimating the carrying capacity of restored areas relative to current population densities (Guisan et al., 2013). Moreover, as species distributions shift rapidly, there is increasing application of dynamic spatially explicit management to mitigate habitat loss and fragmentation, bycatch (unintentionally caught non-target species), human-wildlife interactions, and so forth (Oestreich, Chapman & Crowder, 2020, and references therein). For such dynamic management of features like protected areas, the potential consequences of species distribution modeling choices are similar to those of habitat restoration, but with perhaps greater sensitivity to errors in the location of the core of species distributions. For example, to lower bycatch rates one may attempt to close an area to fishing where the probability of occurrence is highest at any given time, yet sub-optimal location choices could result in costs of greater bycatch and reduced fishing opportunities. As others have demonstrated (*e.g*., Araújo et al., 2005), when the decision of prediction surface resolution does significantly bias predictions, it is likely to have the greatest influence on management success in these two conservation contexts as it influences estimates of local population density at fine scales and coarse-scale summary metrics of distribution (such as the center of gravity in our case) derived from these. While we did not observe this effect of prediction surface resolution on predictions carrying over to estimates of biomass, we did see a slight effect of prediction resolution on center of gravity. Meanwhile, we demonstrated stronger effects of how model structure and data filtering can bias the center of gravity, and how data filtering can increase uncertainty in estimates of this distribution metric. Thus, making the wrong choices within these two decision points could lead to the inference that sablefish are becoming more (less) available to fishing ports in the southern (northern) end of the domain.

### Frontiers in SDM development and exploration

There are a number of frontiers in SDM development and exploration that warrant further examination:

- Spatiotemporal scales: We urge further development of methods for determining appropriate spatial and temporal scales at which to model relationships between responses and predictors, building on studies aimed at evaluating non-local or landscape effects on species distributions (Chandler & Hepinstall-Cymerman, 2016). Such developments are critical for examining local *vs* regional drivers of species distributions.
- Computational: An ongoing frontier in SDM development is computational speed and efficiency. Further development of methods, such as Bayesian sampling approaches (*e.g*., Margossian et al., 2020), that improve computation are critical advancements, especially for working with large datasets (as is common in ecology).
- Data assimilation: Exploring methods for combining multiple data sources (*e.g*., Grüss & Thorson, 2019; Webster et al., 2020; Rufener et al., 2021) in the estimation process is another frontier that will greatly further SDM development. The need for such methods arises commonly because there are limited resources for data collection, sampling methods and intensity change over time (resulting in spatially and temporally imbalanced datasets), and researchers are often interested in phenomena at broader ecological scales than can be evaluated using a single data source. Climate change can exacerbate these challenges and create new ones by, for example, changing the availability of species relative to the spatial extent of surveys. Furthermore, increased data assimilation may improve precision of estimates from SDMs (*e.g*., Grüss & Thorson, 2019).
- Improving projections and quantifying predictive performance: Because of the impacts of climate change, the importance of predicting population responses to novel environments and conditions will only increase. Therefore, further exploration of this topic (*e.g*., Muhling et al., 2020) will become imperative. Furthermore, as SDM use continues to increase, methods for quantifying predictive performance will need to be further developed. In particular, advances in methods for cross-validation of spatial and spatiotemporal models (Roberts et al., 2017; Valavi et al., 2019) will allow computation of the performance metrics that are most suitable for a given application. Such advances could also reveal under what conditions full cross-validation can be adequately replaced using less computationally intensive approaches to select among competing models (*e.g*., AIC).
- Tailoring approaches to specific management applications: Finer-scale analyses of population density like those in this study can help detect or predict local depletion that may otherwise be obscured by coarse-scale or non-spatial resource assessment methods. This is becoming particularly important for managers performing scenario planning of socio-ecological impacts of climate-driven species distribution shifts, as such issues are often lost in the mismatch between scales of climate prediction, regional management, and ecological processes (Holsman et al., 2019). Furthermore, as it is becoming clear that fisheries portfolios (diversification of sampling gear, species targeted, seasons fished) are a key path to maintaining the profitability of fishers in response to environmental variation (*e.g*., Anderson et al., 2017), additional analyses are needed to assess effects of shifting local availability of fishes in space and time (*e.g*., Selden et al., 2020).
- Multi-species distribution models: Species distribution models typically do not explicitly model species interactions, but these relationships greatly shape species distributions (Pendleton et al., 2012; Peoples, Blanc & Frimpong, 2015; Wagner et al., 2020). While joint species distribution models (Thorson & Barnett, 2017; Ovaskainen & Abrego, 2020; Tikhonov et al., 2020; Wagner et al., 2020) can help address this critical methods gap, many current implementations provide only phenomenological representation of associations among species. Thus, there is a particular need for development of mechanistic multi-species SDMs.

## Supplemental Information

10.7717/peerj.12783/supp-1 Supplemental Information 1 Supplemental Figures and Tables.

## Additional Information and Declarations

### Data Availability

The following information was supplied regarding data availability: Additional modeling details and code to replicate analyses are available at GitHub: https://github.com/fate-spatialindicators/shadow-model.

## References

[ref-1] Abrahamsen P (1997). A review of Gaussian random fields and correlation functions. Norwegian Computing Center.

[ref-2] Anderson SC, Ward EJ, Shelton AO, Adkison MD, Beaudreau AH, Brenner RE, Haynie AC, Shriver JC, Watson JT, Williams BC (2017). Benefits and risks of diversification for individual fishers. Proceedings of The National Academy of Sciences.

[ref-3] Anderson SC, Ward EJ (2019). Black swans in space: modeling spatiotemporal processes with extremes. Ecology.

[ref-4] Anderson SC, Keppel EA, Edwards A (2019). A reproducible data synopsis for over 100 species of British Columbia groundfish. DFO Canadian Science Advisory Secretariat Research Document.

[ref-5] Anderson SC, Ward EJ, Barnett LAK, English PA (2020). sdmTMB: spatiotemporal species distribution GLMMs with ‘TMB’. https://github.com/pbs-assess/sdmTMB.

[ref-6] Araújo MB, Thuiller W, Williams PH, Reginster I (2005). Downscaling European species atlas distributions to a finer resolution: implications for conservation planning. Global Ecology and Biogeography.

[ref-7] Bakka H, Rue H, Fuglstad GA, Riebler A, Bolin D, Illian J, Krainski E, Simpson D, Lindgren F (2018). Spatial modeling with R-INLA: a review. Wiley Interdisciplinary Reviews: Computational Statistics.

[ref-8] Barnett LA, Ward EJ, Jannot JE, Shelton AO (2019). Dynamic spatial heterogeneity reveals interdependence of marine faunal density and fishery removals. Ecological Indicators.

[ref-9] Barnett LA, Ward EJ, Anderson SC (2021). Improving estimates of species distribution change by incorporating local trends. Ecography.

[ref-10] Berger AM, Goethel DR, Lynch PD, Quinn T, Mormede S, McKenzie J, Dunn A (2017). Space oddity: the mission for spatial integration. Canadian Journal of Fisheries and Aquatic Sciences.

[ref-11] Breivik ON, Aanes F, Søvik G, Aglen A, Mehl S, Johnsen E (2021). Predicting abundance indices in areas without coverage with a latent spatio-temporal Gaussian model. ICES Journal of Marine Science.

[ref-12] Briscoe NJ, Elith J, Salguero-Gómez R, Lahoz-Monfort JJ, Camac JS, Giljohann KM, Holden MH, Hradsky BA, Kearney MR, McMahon SM, Phillips BL (2019). Forecasting species range dynamics with process-explicit models: matching methods to applications. Ecology Letters.

[ref-13] Brodie SJ, Thorson JT, Carroll G, Hazen EL, Bograd S, Haltuch MA, Holsman KK, Kotwicki S, Samhouri JF, Willis-Norton E, Selden RL (2020). Trade-offs in covariate selection for species distribution models: a methodological comparison. Ecography.

[ref-14] Burrows MT, Schoeman DS, Buckley LB, Moore P, Poloczanska ES, Brander KM, Brown C, Bruno JF, Duarte CM, Halpern BS, Holding J (2011). The pace of shifting climate in marine and terrestrial ecosystems. Science.

[ref-15] Chandler R, Hepinstall-Cymerman J (2016). Estimating the spatial scales of landscape effects on abundance. Landscape Ecology.

[ref-16] Chen IC, Hill JK, Ohlemüller R, Roy DB, Thomas CD (2011). Rapid range shifts of species associated with high levels of climate warming. Science.

[ref-17] Cheung WW, Lam VW, Sarmiento JL, Kearney K, Watson R, Pauly D (2009). Projecting global marine biodiversity impacts under climate change scenarios. Fish and Fisheries.

[ref-18] Chuine I (2010). Why does phenology drive species distribution?. Philosophical Transactions of the Royal Society B: Biological Sciences.

[ref-19] Connor T, Viña A, Winkler JA, Hull V, Tang Y, Shortridge A, Yang H, Zhao Z, Wang F, Zhang J, Zhang Z (2019). Interactive spatial scale effects on species distribution modeling: the case of the giant panda. Scientific Reports.

[ref-20] Cotton PA (2003). Avian migration phenology and global climate change. Proceedings of The National Academy of Sciences.

[ref-21] Cressie N, Wikle CK (2015). Statistics for spatio-temporal data.

[ref-22] Distler T, Schuetz JG, Velásquez-Tibatá J, Langham GM (2015). Stacked species distribution models and macroecological models provide congruent projections of avian species richness under climate change. Journal of Biogeography.

[ref-23] Doney SC, Ruckelshaus M, Emmett Duffy J, Barry JP, Chan F, English CA, Galindo HM, Grebmeier JM, Hollowed AB, Knowlton N, Polovina J (2012). Climate change impacts on marine ecosystems. Annual Review of Marine Science.

[ref-24] Draper D, Krnjajic M (2010). Calibration results for Bayesian model specification. Bayesian Analysis.

[ref-25] Dunn PK, Smyth GK (1996). Randomized quantile residuals. Journal of Computational and Graphical Statistics.

[ref-26] Dunn PK, Smyth GK (2005). Series evaluation of Tweedie exponential dispersion model densities. Statistics and Computing.

[ref-27] Dunn PK, Smyth GK (2008). Evaluation of Tweedie exponential dispersion model densities by Fourier inversion. Statistics and Computing.

[ref-28] Edwards M, Richardson AJ (2004). Impact of climate change on marine pelagic phenology and trophic mismatch. Nature.

[ref-30] Elith JH, Graham CP, Anderson R, Dudík M, Ferrier S, Guisan A, Hijmans RJ, Huettmann F, Leathwick JR, Lehmann A, Li J (2006). Novel methods improve prediction of species’ distributions from occurrence data. Ecography.

[ref-31] Elith J, Leathwick JR, Hastie T (2008). A working guide to boosted regression trees. Journal of Animal Ecology.

[ref-32] Elith J, Leathwick JR (2009). Species distribution models: ecological explanation and prediction across space and time. Annual Review of Ecology, Evolution, and Systematics.

[ref-33] Elith J, Phillips SJ, Hastie T, Dudík M, Chee YE, Yates CJ (2011). A statistical explanation of MaxEnt for ecologists. Diversity and Distributions.

[ref-29] English PA, Ward EJ, Rooper CN, Forrest RE, Rogers LA, Hunter KL, Edwards AM, Connors BM, Anderson SC (2022). Contrasting climate velocity impacts in warm and cool locations show that effects of marine warming are worse in already warmer temperate waters. Fish and Fisheries.

[ref-34] Eschmeyer WN, Herald ES (1999). A field guide to Pacific coast fishes: North America.

[ref-35] Foley JA, DeFries R, Asner GP, Barford C, Bonan G, Carpenter SR, Chapin FS, Coe MT, Daily GC, Gibbs HK, Helkowski JH (2005). Global consequences of land use. Science.

[ref-36] Francis RC (2011). Data weighting in statistical fisheries stock assessment models. Canadian Journal of Fisheries and Aquatic Sciences.

[ref-37] Goethel DR, Quinn TJ, Cadrin SX (2011). Incorporating spatial structure in stock assessment: movement modeling in marine fish population dynamics. Reviews in Fisheries Science.

[ref-38] Grüss A, Thorson JT (2019). Developing spatio-temporal models using multiple data types for evaluating population trends and habitat usage. ICES Journal of Marine Science.

[ref-39] Guillera-Arroita G, Lahoz-Monfort JJ, Elith J, Gordon A, Kujala H, Lentini PE, McCarthy MA, Tingley R, Wintle BA (2015). Is my species distribution model fit for purpose? Matching data and models to applications. Global Ecology and Biogeography.

[ref-40] Guisan A, Edwards TC, Hastie T (2002). Generalized linear and generalized additive models in studies of species distributions: setting the scene. Ecological Modelling.

[ref-41] Guisan A, Graham CH, Elith J, Huettmann F, the NCEAS Species Distribution Modelling Group (2007). Sensitivity of predictive species distribution models to change in grain size. Diversity and Distributions.

[ref-42] Guisan A, Tingley R, Baumgartner JB, Naujokaitis-Lewis I, Sutcliffe PR, Tulloch AI, Regan TJ, Brotons L, McDonald-Madden E, Mantyka-Pringle C, Martin TG (2013). Predicting species distributions for conservation decisions. Ecology Letters.

[ref-43] Haltuch MA, Johnson KF, Tolimieri N, Kapur MS, Castillo-Jordán CA (2019). Status of the sablefish stock in US waters in 2019.

[ref-44] Hamel OS (2007). Status and future prospects for the darkblotched rockfish resource in waters off Washington, Oregon and California as assessed in 2007.

[ref-45] Hartig F (2021). DHARMa: residual diagnostics for hierarchical multi-level and mixed regression models. http://florianhartig.github.io/DHARMa/.

[ref-46] Hickling R, Roy DB, Hill JK, Fox R, Thomas CD (2006). The distributions of a wide range of taxonomic groups are expanding polewards. Global Change Biology.

[ref-47] Hilborn R, Walters CJ (1992). Quantitative fisheries stock assessment: choice, dynamics and uncertainty.

[ref-48] Holsman KK, Hazen EL, Haynie A, Gourguet S, Hollowed A, Bograd SJ, Samhouri JF, Aydin K (2019). Towards climate resiliency in fisheries management. ICES Journal of Marine Science.

[ref-49] Jarnevich CS, Stohlgren TJ (2009). Near term climate projections for invasive species distributions. Biological Invasions.

[ref-50] Jiménez-Valverde A (2012). Insights into the area under the receiver operating characteristic curve (AUC) as a discrimination measure in species distribution modelling. Global Ecology and Biogeography.

[ref-51] Johnson KF, Thorson JT, Punt AE (2019). Investigating the value of including depth during spatiotemporal index standardization. Fisheries Research.

[ref-52] Keller AA, Wallace JR, Methot RD (2017). The Northwest Fisheries Science Center’s West Coast Groundfish Bottom Trawl Survey: history, design, and description. https://repository.library.noaa.gov/view/noaa/14179.

[ref-53] Kelly AE, Goulden ML (2008). Rapid shifts in plant distribution with recent climate change. Proceedings of The National Academy of Sciences.

[ref-54] Kristensen K, Nielsen A, Berg CW, Skaug HJ, Bell B (2016). TMB: automatic differentiation and laplace approximation. Journal of Statistical Software.

[ref-55] Latimer AM, Banerjee S, Sang H, Mosher ES, Silander JA (2009). Hierarchical models facilitate spatial analysis of large data sets: a case study on invasive plant species in the northeastern United States. Ecology Letters.

[ref-56] Leidenberger S, De Giovanni R, Kulawik R, Williams AR, Bourlat SJ (2015). Mapping present and future potential distribution patterns for a meso-grazer guild in the Baltic Sea. Journal of Biogeography.

[ref-57] Levinsky I, Skov F, Svenning JC, Rahbek C (2007). Potential impacts of climate change on the distributions and diversity patterns of European mammals. Biodiversity and Conservation.

[ref-58] Liang H, Wu H, Zou G (2008). A note on conditional AIC for linear mixed-effects models. Biometrika.

[ref-59] Lindgren F, Rue H, Lindström J (2011). An explicit link between Gaussian fields and Gaussian Markov random fields: the stochastic partial differential equation approach. Journal of the Royal Statistical Society: Series B (Statistical Methodology).

[ref-60] Lindgren F, Rue H (2015). Bayesian spatial modelling with R-INLA. Journal of Statistical Software.

[ref-61] Lowen JB, McKindsey CW, Therriault TW, DiBacco C (2016). Effects of spatial resolution on predicting the distribution of aquatic invasive species in nearshore marine environments. Marine Ecology Progress Series.

[ref-62] Margossian C, Vehtari A, Simpson D, Agrawal R (2020). Hamiltonian Monte Carlo using an adjoint-differentiated Laplace approximation: Bayesian inference for latent Gaussian models and beyond.

[ref-63] Martínez-Minaya J, Cameletti M, Conesa D, Pennino MG (2018). Species distribution modeling: a statistical review with focus in spatio-temporal issues. Stochastic Environmental Research and Risk Assessment.

[ref-64] Matthews SN, Iverson LR, Prasad AM, Peters MP (2011). Changes in potential habitat of 147 North American breeding bird species in response to redistribution of trees and climate following predicted climate change. Ecography.

[ref-65] McCullagh P, Nelder J (1989). Generalized linear models.

[ref-66] Merow C, Smith MJ, Silander JA (2013). A practical guide to MaxEnt for modeling species’ distributions: what it does, and why inputs and settings matter. Ecography.

[ref-67] Methot RD, Stewart IJ (2005). Status of the US canary rockfish resource in 2005.

[ref-68] Miller DL, Glennie R, Seaton AE (2020). Understanding the stochastic partial differential equation approach to smoothing. Journal of Agricultural, Biological and Environmental Statistics.

[ref-69] Morley JW, Selden RL, Latour RJ, Frölicher TL, Seagraves RJ, Pinsky ML (2018). Projecting shifts in thermal habitat for 686 species on the North American continental shelf. PLOS ONE.

[ref-70] Muhling BA, Brodie S, Smith JA, Tommasi D, Gaitan CF, Hazen EL, Jacox MG, Auth TD, Brodeur RD (2020). Predictability of species distributions deteriorates under novel environmental conditions in the California Current System. Frontiers in Marine Science.

[ref-71] Muscatello A, Elith J, Kujala H (2021). How decisions about fitting species distribution models affect conservation outcomes. Conservation Biology.

[ref-72] Norberg A, Abrego N, Blanchet FG, Adler FR, Anderson BJ, Anttila J, Araújo MB, Dallas T, Dunson D, Elith J, Foster SD (2019). A comprehensive evaluation of predictive performance of 33 species distribution models at species and community levels. Ecological Monographs.

[ref-73] Oremus KL, Bone J, Costello C, Molinos JG, Lee A, Mangin T, Salzman J (2020). Governance challenges for tropical nations losing fish species due to climate change. Nature Sustainability.

[ref-74] Osgood-Zimmerman A, Wakefield J (2021). A statistical introduction to template model builder: a flexible tool for spatial modeling. https://arxiv.org/abs/2103.09929.

[ref-75] Oestreich WK, Chapman MS, Crowder LB (2020). A comparative analysis of dynamic management in marine and terrestrial systems. Frontiers in Ecology and the Environment.

[ref-76] Ovaskainen O, Abrego N (2020). Joint species distribution modelling: with applications in R.

[ref-77] Paradinas I, Conesa D, López-Quílez A, Esteban A, López LM, Bellido JM, Pennino MG (2020). Assessing the spatiotemporal persistence of fish distributions: a case study on two red mullet species (*Mullus surmuletus* and *M. barbatus*) in the western Mediterranean. Marine Ecology Progress Series.

[ref-78] Parmesan C (2006). Ecological and evolutionary responses to recent climate change. Annual Review of Ecology, Evolution, and Systematics.

[ref-79] Pearson RG, Raxworthy CJ, Nakamura M, Townsend Peterson A (2007). Predicting species distributions from small numbers of occurrence records: a test case using cryptic geckos in Madagascar. Journal of Biogeography.

[ref-80] Pendleton RM, Pritt JJ, Peoples BK, Frimpong EA (2012). The strength of Nocomis nest association contributes to patterns of rarity and commonness among New River, Virginia cyprinids. The American Midland Naturalist.

[ref-81] Pennington M (1983). Efficient estimators of abundance, for fish and plankton surveys. Biometrics.

[ref-82] Peoples BK, Blanc LA, Frimpong EA (2015). Lotic cyprinid communities can be structured as nest webs and predicted by the stress-gradient hypothesis. Journal of Animal Ecology.

[ref-83] Perry AL, Low PJ, Ellis JR, Reynolds JD (2005). Climate change and distribution shifts in marine fishes. Science.

[ref-84] Poloczanska ES, Burrows MT, Brown CJ, García Molinos J, Halpern BS, Hoegh-Guldberg O, Kappel CV, Moore PJ, Richardson AJ, Schoeman DS, Sydeman WJ (2016). Responses of marine organisms to climate change across oceans. Frontiers in Marine Science.

[ref-85] Porfirio LL, Harris RM, Lefroy EC, Hugh S, Gould SF, Lee G, Bindoff NL, Mackey B (2014). Improving the use of species distribution models in conservation planning and management under climate change. PLOS ONE.

[ref-86] R Core Team (2020). *R: a language and environment for statistical computing*.

[ref-87] Righetto AJ, Faes C, Vandendijck Y, Ribeiro PJ (2018). On the choice of the mesh for the analysis of geostatistical data using R-INLA. Communications in Statistics-Theory and Methods.

[ref-88] Roberts DR, Bahn V, Ciuti S, Boyce MS, Elith J, Guillera-Arroita G, Hauenstein S, Lahoz-Monfort JJ, Schröder B, Thuiller W, Warton DI (2017). Cross-validation strategies for data with temporal, spatial, hierarchical, or phylogenetic structure. Ecography.

[ref-89] Robinson NM, Nelson WA, Costello MJ, Sutherland JE, Lundquist CJ (2017). A systematic review of marine-based species distribution models (SDMs) with recommendations for best practice. Frontiers in Marine Science.

[ref-90] Rue H, Martino S, Chopin N (2009). Approximate Bayesian inference for latent Gaussian models by using integrated nested Laplace approximations. Journal of the Royal Statistical Society: Series B.

[ref-91] Rufener MC, Kristensen K, Nielsen JR, Bastardie F (2021). Bridging the gap between commercial fisheries and survey data to model the spatiotemporal dynamics of marine species. Ecological Applications.

[ref-92] Scales KL, Hazen EL, Jacox MG, Edwards CA, Boustany AM, Oliver MJ, Bograd SJ (2017). Scale of inference: on the sensitivity of habitat models for wide-ranging marine predators to the resolution of environmental data. Ecography.

[ref-93] Selden RL, Thorson JT, Samhouri JF, Bograd SJ, Brodie S, Carroll G, Haltuch MA, Hazen EL, Holsman KK, Pinsky ML, Tolimieri N (2020). Coupled changes in biomass and distribution drive trends in availability of fish stocks to US West Coast ports. ICES Journal of Marine Science.

[ref-94] Shelton AO, Thorson JT, Ward EJ, Feist BE (2014). Spatial semiparametric models improve estimates of species abundance and distribution. Canadian Journal of Fisheries and Aquatic Sciences.

[ref-95] Stock BC, Ward EJ, Eguchi T, Jannot JE, Thorson JT, Feist BE, Semmens BX (2020). Comparing predictions of fisheries bycatch using multiple spatiotemporal species distribution model frameworks. Canadian Journal of Fisheries and Aquatic Sciences.

[ref-96] Thorson JT, Ward EJ (2013). Accounting for space-time interactions in index standardization models. Fisheries Research.

[ref-97] Thorson JT, Shelton AO, Ward EJ, Skaug HJ (2015). Geostatistical delta-generalized linear mixed models improve precision for estimated abundance indices for West Coast groundfishes. ICES Journal of Marine Science.

[ref-99] Thorson JT, Pinsky ML, Ward EJ (2016). Model-based inference for estimating shifts in species distribution, area occupied and centre of gravity. Methods in Ecology and Evolution.

[ref-100] Thorson JT, Barnett LA (2017). Comparing estimates of abundance trends and distribution shifts using single-and multispecies models of fishes and biogenic habitat. ICES Journal of Marine Science.

[ref-101] Thorson JT (2019). Guidance for decisions using the Vector Autoregressive Spatio-Temporal (VAST) package in stock, ecosystem, habitat and climate assessments. Fisheries Research.

[ref-102] Thorson JT, Cunningham CJ, Jorgensen E, Havron A, Hulson PJ, Monnahan CC, von Szalay P (2021). The surprising sensitivity of index scale to delta-model assumptions: recommendations for model-based index standardization. Fisheries Research.

[ref-103] Thygesen UH, Albertsen CM, Berg CW, Kristensen K, Nielsen A (2017). Validation of ecological state space models using the Laplace approximation. Environmental and Ecological Statistics.

[ref-104] Tikhonov G, Opedal ØH, Abrego N, Lehikoinen A, de Jonge MM, Oksanen J, Ovaskainen O (2020). Joint species distribution modelling with the r-package Hmsc. Methods in Ecology and Evolution.

[ref-105] Tolimieri N (2007). Patterns in species richness, species density, and evenness in groundfish assemblages on the continental slope of the US Pacific coast. Environmental Biology of Fishes.

[ref-106] Tolimieri N, Haltuch MA, Lee Q, Jacox MG, Bograd SJ (2018). Oceanographic drivers of sablefish recruitment in the California Current. Fisheries Oceanography.

[ref-107] Tredennick AT, Hooker G, Ellner SP, Adler PB (2021). A practical guide to selecting models for exploration, inference, and prediction in ecology. Ecology.

[ref-108] Valavi R, Elith J, Lahoz-Monfort JJ, Guillera-Arroita G (2019). Block CV: an R package for generating spatially or environmentally separated folds for k-fold cross-validation of species distribution models. Methods in Ecology and Evolution.

[ref-109] Ver Hoef JM, Hanks EM, Hooten MB (2018). On the relationship between conditional (CAR) and simultaneous (SAR) autoregressive models. Spatial Statistics.

[ref-110] Wagner T, Hansen GJ, Schliep EM, Bethke BJ, Honsey AE, Jacobson PC, Kline BC, White SL (2020). Improved understanding and prediction of freshwater fish communities through the use of joint species distribution models. Canadian Journal of Fisheries and Aquatic Sciences.

[ref-111] Wall MM (2004). A close look at the spatial structure implied by the CAR and SAR models. Journal of Statistical Planning and Inference.

[ref-112] Walther GR (2010). Community and ecosystem responses to recent climate change. Philosophical Transactions of the Royal Society B: Biological Sciences.

[ref-113] Ward EJ, Oken KL, Rose KA, Sable S, Watkins K, Holmes EE, Scheuerell MD (2018). Applying spatiotemporal models to monitoring data to quantify fish population responses to the Deepwater Horizon oil spill in the Gulf of Mexico. Environmental Monitoring and Assessment.

[ref-114] Webster RA, Soderlund E, Dykstra CL, Stewart IJ (2020). Monitoring change in a dynamic environment: spatiotemporal modelling of calibrated data from different types of fisheries surveys of Pacific halibut. Canadian Journal of Fisheries and Aquatic Sciences.

[ref-115] Woillez M, Rivoirard J, Petitgas P (2009). Notes on survey-based spatial indicators for monitoring fish populations. Aquatic Living Resources.

[ref-116] Yoklavich MM, Love MS, Forney KA (2007). A fishery-independent assessment of an overfished rockfish stock, cowcod (*Sebastes levis*), using direct observations from an occupied submersible. Canadian Journal of Fisheries and Aquatic Sciences.

[ref-117] Zeileis A, Kleiber C, Jackman S (2008). Regression models for count data in R. Journal of Statistical Software.

