# Supplementary material for: The shadow model: how and why small choices in spatially explicit species distribution models affect predictions
Source: PeerJ. 2022 Feb 14;10:e12783. doi: 10.7717/peerj.12783 (PMC8852273; doi:10.7717/peerj.12783)

Supplementary Material for

**The shadow model: how and why small choices in spatially explicit species distribution models affect predictions**

Christian J. C. Commander^*^, Lewis A. K. Barnett, Eric J. Ward, Sean C. Anderson, Timothy E. Essington

*****Corresponding author: ccommander@fsu.edu

**Tables**

Table S1. Annual relative sablefish biomass estimates and coefficients of variation (CV) for fine and coarse (4x) prediction surface resolutions.

|  | **Fine Resolution** | | **Coarse Resolution** | |
| --- | --- | --- | --- | --- |
| ***Year*** | ***Relative Biomass*** | ***CV*** | ***Relative Biomass*** | ***CV*** |
| 2003 | 1.000 | 0.003 | 1.000 | 0.004 |
| 2004 | 0.917 | 0.004 | 0.917 | 0.005 |
| 2005 | 0.765 | 0.004 | 0.765 | 0.004 |
| 2006 | 0.687 | 0.004 | 0.687 | 0.004 |
| 2007 | 0.593 | 0.003 | 0.593 | 0.004 |
| 2008 | 0.431 | 0.004 | 0.431 | 0.005 |
| 2009 | 0.479 | 0.004 | 0.479 | 0.005 |
| 2010 | 0.462 | 0.004 | 0.462 | 0.004 |
| 2011 | 0.550 | 0.004 | 0.550 | 0.004 |
| 2012 | 0.456 | 0.004 | 0.456 | 0.005 |
| 2013 | 0.505 | 0.004 | 0.505 | 0.005 |
| 2014 | 0.660 | 0.004 | 0.660 | 0.004 |
| 2015 | 0.566 | 0.004 | 0.566 | 0.004 |
| 2016 | 0.558 | 0.004 | 0.558 | 0.004 |
| 2017 | 0.784 | 0.003 | 0.784 | 0.004 |
| 2018 | 0.919 | 0.003 | 0.919 | 0.004 |
|  |  |  |  |  |
| ***Mean*** |  | 0.0036 |  | 0.0044 |

Table S2. Sablefish center of gravity (COG) with lower and upper 95% confidence intervals for fine and coarse (4x) prediction surface resolutions.

|  | **Prediction Grid Resolution** | |
| --- | --- | --- |
|  | ***Fine*** | ***Coarse*** |
| ***Northings (km)*** | 4532.626 | 4515.091 |
| ***Northings Lower*** | 4512.964 | 4489.654 |
| ***Northings Upper*** | 4552.289 | 4540.527 |
| ***Northings Diameter*** | 39.325 | 50.874 |
|  |  |  |
| ***Eastings (km)*** | 461.699 | 465.792 |
| ***Eastings Lower*** | 455.565 | 457.549 |
| ***Eastings Upper*** | 467.834 | 474.035 |
| ***Eastings Diameter*** | 12.269 | 16.487 |

**Figures**

Figure S1. Meshes with 50, 300, and 600 knots. The dots are data observations.


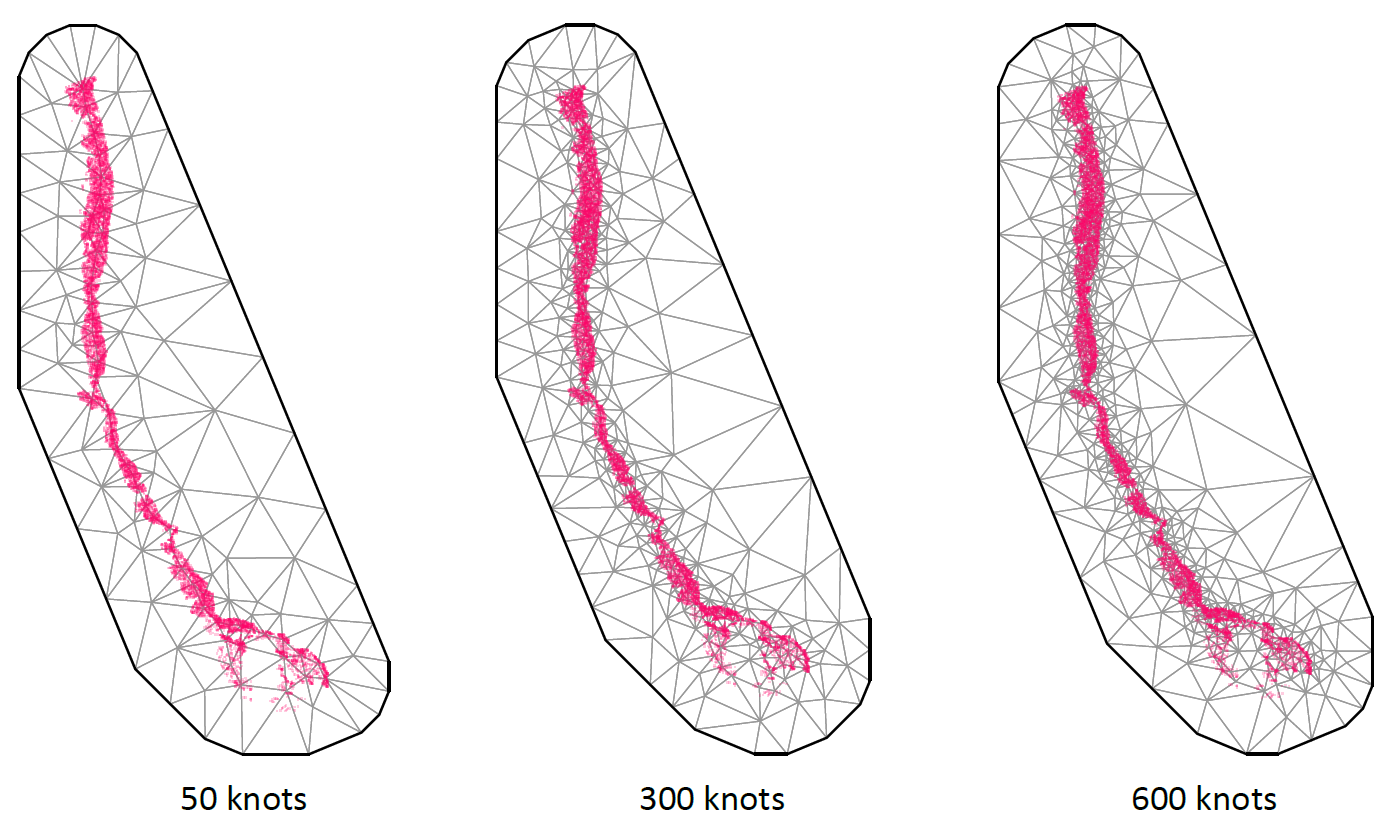


Figure S2. Q-Q plots using simulation-based residuals (DHARMa) comparing a spatial only model, a spatial model with depth covariate, and a spatiotemporal (IID) model with depth covariate.


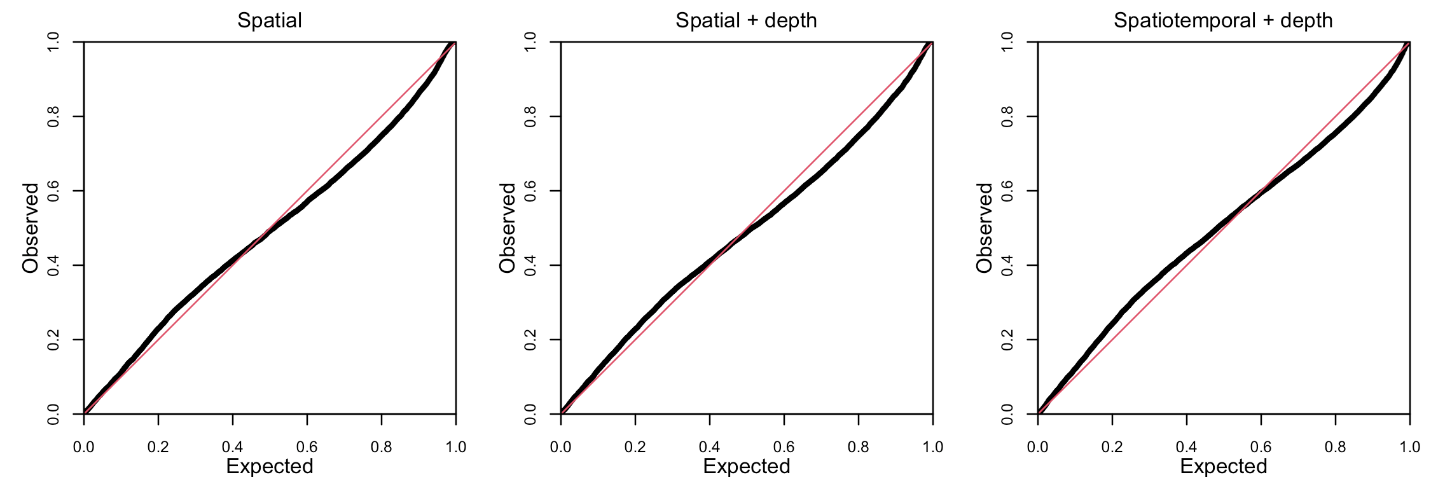


Figure S3. Relative sablefish biomass estimates (standardized to each time series maximum estimate) for each year (with 95% confidence intervals) for fine and coarse (4x) prediction surface resolutions.


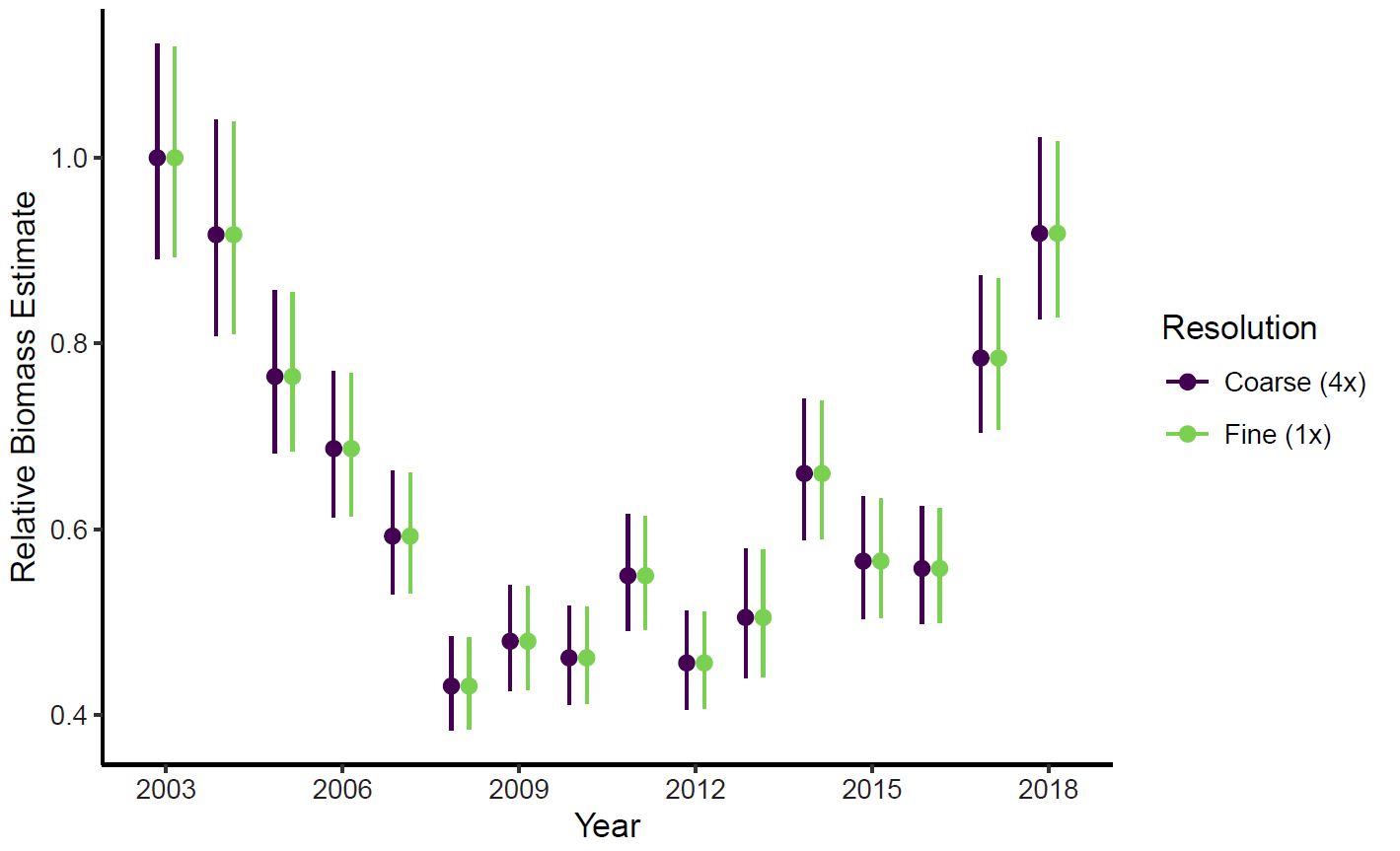

Supplement: Supplemental Information 1 [file peerj-10-12783-s001.docx]
